# Patients with Inflammatory Bowel Disease Exhibit Dysregulated Responses to Microbial DNA

**DOI:** 10.1371/journal.pone.0037932

**Published:** 2012-05-23

**Authors:** Naomi S. C. Hotte, Saad Y. Salim, Robert H. Tso, Eric J. Albert, Phil Bach, John Walker, Levinus A. Dieleman, Richard N. Fedorak, Karen L. Madsen

**Affiliations:** Department of Medicine, University of Alberta, Edmonton, Alberta, Canada; Charité-University Medicine Berlin, Germany

## Abstract

**Background:**

A critical role for the gut epithelium lies in its ability to discriminate between pathogens and commensals and respond appropriately. Dysfunctional interactions between microbes and epithelia are believed to have a role in inflammatory bowel disease (IBD). In this study, we analyzed microbiota and gene expression in IBD patients and examined responses of mucosal biopsies to bacterial DNA.

**Methods:**

Biopsies were taken from non-inflamed areas of the colon in healthy controls (HC) and Crohn's disease (CD) and ulcerative colitis (UC) patients in remission. Biopsies were snap-frozen or cultured with DNA from *Lactobacillus plantarum* (LP) or *Salmonella dublin* (SD). Gene expression was analyzed under basal conditions and in response to DNA. Gene networks were analyzed using Ingenuity Pathways software. Mucosal-associated microbiota was analyzed using terminal restriction fragment length polymorphism. Frequency of single nucleotide polymorphisms in NOD2 and TLR9 was assessed.

**Results:**

Patients with IBD had altered microbiota, enhanced expression of inflammatory genes, and increased correlations between specific gene expression and microbes. Principle component analysis showed CD and UC patients to cluster independently from healthy controls in both gene expression and microbial analysis. DNA from LP stimulated anti-inflammatory pathways in controls and UC patients, but induced an upregulation of IL17A in CD patients. There were no differences in SNP frequencies of TLR9 or NOD2 in the groups.

**Conclusions:**

Patients with Crohn's disease exhibit altered responses to bacterial DNA. These findings suggest that the gut response to bacterial DNA may depend not only on the specific type of bacterial DNA, but also on the host.

## Introduction

Inflammatory bowel diseases, including Crohn's disease (CD) and ulcerative colitis (UC), are chronic relapsing disorders that are thought to occur as a result of a loss of tolerance to normal commensal microbiota [1]. The recent discoveries of a role for NOD2 and ATG16L1 genes as risk factors have emphasized how defects in the innate recognition and response to microbial compounds can influence disease and result in immune dysregulation and microbial dysbiosis. Patients with CD exhibit a decrease in bacterial diversity and a dysbiosis with reduced amounts of protective strains such as *Faecalibacterium prausnitzii* [2] and increased levels of inflammatory strains such as adherent invasive *E. coli* [3]–[6]. While the role for intestinal bacteria in the pathogenesis of IBD is strongly suggested by clinical and experimental evidence, it is equally clear that not all bacteria induce intestinal inflammatory responses and that some strains, such as *F. prausnitzii*, can actually reduce and modulate intestinal inflammation [2]. The use of specific strains of probiotics to modulate and reduce gut inflammation in patients with IBD has resulted in positive clinical trials for UC, but interestingly, not for CD [7]. The reason for this is currently unknown; however, it is possible that either the genetic background and/or an altered luminal environment might significantly alter the gut response to probiotics.

In the gut, bacterial DNA is recognized by toll-like receptor 9 (TLR9) on epithelial and immune cells and by the intracellular inflammasome. TLR9 is located on the apical and the basolateral membrane of epithelial cells and cellular responses to bacterial DNA are dependent upon both the site of stimulation as well as by the CpG sequences [8], [9]. We have previously shown that stimulation of intestinal epithelial cells with bacterial DNA from a pathogenic strain such as *Salmonella dublin* results in an inflammatory response and enhanced secretion of IL-8, while bacterial DNA from commensal or probiotic strains elicits no response [9]. Additionally, we have shown in an *in vitro* model that the presence of pro-inflammatory cytokines can significantly alter epithelial and immune cell responses to bacterial DNA [10], suggesting a role for environmental factors in modulating TLR9 signaling. In numerous studies, anti-inflammatory effects of probiotics have been linked with TLR9 signaling in the gut, suggesting a dominant role for TLR9 and bacterial DNA in mediating effects of probiotics [11], [12]. In that IBD patients have both altered gut microbiota and an inflammatory milieu within the lamina propria, we hypothesized that IBD patients would not respond to bacterial DNA in a similar fashion as healthy controls. To test this hypothesis, we characterized the gut microenvironment with regards to basal gene expression and mucosal-associated microbiota in colonic biopsies from IBD patients and analyzed the tissue response to probiotic and pathogenic bacterial DNA. In support of our hypothesis, we show different gene networks are stimulated in IBD patients in response to bacterial DNA compared with healthy controls, and further, that these differences are associated with both altered gut microbiota and basal gene expression.

## Methods

### Patient Population

Biopsies were obtained from macroscopically normal areas of the transverse colon in patients with endoscopic and histologic confirmed diagnosis of UC for at least one year, or patients with a similar diagnosis of CD of at least three months' duration. Patients were excluded if they had a history of dysplasia of the colon or any cancer in the last five years, serious underlying disease other than UC/CD, and/or severely impaired liver or renal function. Biopsies from healthy controls were obtained from patients undergoing colonoscopy for screening purposes. Biopsies were either frozen immediately or placed in 0.5 ml of sterile cell culture media and transferred to an incubator. Adjacent biopsies were taken for routine histopathological examination. All patients were informed about the study and provided written consent. The study was approved by the University of Alberta ethics committee (Pro00001799).

### Bacterial strains and Preparation of DNA

*Salmonella dublin* strain Lane (ATCC #15480) was chosen as a representative pathogen and *Lactobacillus plantarum* MB 452 (VSL#3 Pharmaceuticals) as a representative probiotic strain for these studies as we have previously shown significantly different responses to isolated DNA from these strains in cell culture models [9]. Strains were grown overnight at 37°C under aerobic conditions in Luria-burtini (LB) broth (BD 244620) and under anaerobic conditions in Lactobacilli MRS broth (BD 288130), respectively. DNA was isolated as previously described [9].

### Culture of Biopsies

Whole-thickness biopsies (5–10 mg) were placed in culture filter plates at 37°C in 1 ml of RPMI 1640 media (100 U/ml penicillin, 100 ug/ml streptomycin, and 50 ug/ml gentamycin) and cultured for 2 hours±50 ug/ml DNA isolated from *Salmonella dublin* or *Lactobacillus plantarum*. After incubation, tissues were harvested in RNAlater and stored at −80°C.

### Microbial Analysis

Microbes associated with the biopsies were assessed using terminal restriction fragment length polymorphism (T-RFLP). Total DNA was extracted from biopsies using a FastDNA Spin Kit (MP Biomedical) as per manufacturer's instructions. 16S rRNA was amplified by PCR using a 6-FAM-5′-labelled, broad-range forward primer 6-FAM-8F (Applied Biosystems), 5′-AGAGTTTGATCCTGGCTCAG-3′) and a broad-range reverse primer 926R (Applied Biosystems) (5′-AGAAAGGAGGTGATCCAGCC-3′). PCR was performed with 50 ng DNA. Cycling conditions consisted of an initial denaturing step at 94°C for 2 min followed by 35 cycles of 94°C 1 min, 56°C 1 min, 72°C 1 min, and a final 10 min extension at 72°C. A DNA-free template control was included in every PCR run and amplification confirmed by visualization of a single 920 kb PCR product on a 1% agarose gel. Amplicons were purified using Qiagen MinElute PCR Purification Kit as per the manufacturer's instructions. Amplicon DNA (200–300 ng, as determined by Nanodrop spectrophotometer measurement (Thermo Scientific, Wilmington, Delaware, USA) was digested with the Hpall restriction enzyme (Promega, Madison, Wisconsin, USA) for 16 hours at 37°C. For each sample, 100 ng of HPAII digested fragments were resolved in duplicate using a 3130XL Genetic Analyzer (Applied Biosystems, Carlsbad, California, USA). Each sample was separated with an internal ROX1000 DNA marker to enable fragment length normalization. Bionumerics 6.0 software (Applied Maths, Sint-Martens-Latem, Belgium) was used to normalize fluorescently labeled terminal fragment lengths and select peaks of interest. Selected peaks of interest were associated, *in silico*, with fragment lengths of known bacteria using Microbial Community Analysis 3 (MiCA; Shyu, 2007) and Ribosomal Database Project v.9 (RDP; Cole, 2009). Peaks corresponding to fragments between 25 and 650 base pairs (bp) in length were used in the community composition and cluster analyses. Principal component and clustering analyses were done to map each individual patient based upon their microbial profile and to define specific clusters.

### TaqMan Low Density Array (TLDA) and Correlation Analysis

Total RNA was isolated from cultured and snap-frozen biopsies using a modified TRIzol extraction (Invitrogen, USA) followed by an extra purification using RNeasy columns (Qiagen, USA). Briefly, tissue was homogenised in 1 ml TRIzol then mixed with 200 µl of chloroform and centrifuged at 14000 rpm to separate the aqueous layer. This RNA-containing layer was doubled in volume with 70% ethanol and applied to an RNeasy column by centrifugation as per manufacturer's instructions. Total RNA quantity and integrity were evaluated using a nanodrop 1000 spectrophotometer (Thermo Scientific) and Flashgel system (Lonza, Basel, Switzerland). Relative gene expression was analyzed using 96-plex Human Immune TaqMan Low Density Arrays (TLDA)(Applied Biosystems). cDNA was created using random hexamers and the High Capacity cDNA Reverse Transcription Kit (Applied Biosystems) as per manufacturer's instructions. The relationship between host gene expression and microflora was investigated using Spearman rank correlations. Correlations with an FDR≤5% were considered significant.

### Single nucleotide polymorphisms

The presence of single nucleotide polymorphisms (SNPs) in NOD2 and TLR9 was assessed [13], [14]. Three SNPs were analysed using either single-direction-sequencing for TLR9-1237T/C (rs5743836) and NOD2 SNP13 3020insC (rs2066847) or using SNaPshot Multiplex system (Applied Biosystems) for NOD2 SNP8 2104C/T. Primer sequences are described in Table S1.

### Gene networks

In order to determine biological relevance from the gene expression data, probable gene networks were analyzed using the Ingenuity Pathways Analysis application (Ingenuity Systems, Redwood City, CA). The genes considered were those that were differentially expressed (Fold Change >1.5 or p<0.01) within each treatment group when compared to the control. The relationship between the genes, which are represented by nodes, is denoted by an edge. Red and green nodes represent up- and down-regulated gene expression respectively. Direct interactions between genes are represented by solid lines.

### Statistical analysis

For the study groups, continuous variables were analyzed using t tests. Tests for normality (Kolmogorov–Smirnov) were applied and median values and non-parametric tests (Mann–Whitney U) were used for data that were not normally distributed. For dichotomous variables, differences between groups were compared using χ^2^ or Fisher exact tests.

## Results

### Demographic and clinical data of the patient cohort

Biopsies were obtained from adult patients with CD (n = 15), UC (n = 14) or healthy controls (n = 21) (Table 1). There were no significant differences between the groups in age, gender, or disease duration. All IBD patients were currently in remission. The majority of the IBD patients were receiving drug therapy, including 5-ASA, antibiotics, steroids, immunomodulators, or biologics.

**Table 1:** Clinical parameters of patients.

Category	Sub-Category	Control (n = 21)	Crohn's Disease (n = 15)	Ulcerative Colitis (n = 14)
Mean Age (yrs)(range)		45.6(20–81)	38.3(21–54)	46(20–73)
Gender	Female	8 (38%)	4 (27%)	4 (29%)
	Male	13 (62%)	11 (73%)	10 (71%)
Disease Site	Colonic	n/a	4 (27%)	14 (100%)
	Ileocolonic		5 (33%)	
	Ileal		4 (27%)	
	Undetermined		2 (13%)	
Disease Duration (yrs)		n/a	12 (1–24)	15 (1–33)
Medication	5-ASA	n/a	4 (27%)	11 (79%)
	Steroids		4 (27%)	4 (29%)
	Immunomodulators*		6 (40%)	4 (29%)
	Biologics		4 (27%)	0 (0%)
	Antibiotics		0 (0%)	0 (0%)
	None		1 (7%)	1 (7%)

* Immunomodulators: azathioprine and/or infliximab.

5-ASA- 5-amino salicylic acid.

### Basal Gene Expression and Microflora Composition

Gene expression and microbial composition were analyzed in snap-frozen colonic biopsies in order to determine if the microenvironment differed in control tissue compared with macroscopically non-inflamed tissue from CD and UC patients. Analysis of mucosa-associated microbiota showed no significant differences in the main phyla between the groups (Table 2). However, altered microbial composition within phyla (Table 2) and differentially expressed genes (fold-change ≥1.5) (Table 3) were identified between the healthy controls and patients with CD and UC.

**Table 2:** Microbial analysis of biopsies from healthy controls and CD and UC patients.

Phyla	Class	Control (n = 21)	CD(n = 15)	UC(n = 14)
Firmicutes	% of Total	70±10 (49–91)	71±11 (49–89)	73±12 (55–94)
	Clostridia*	93±5 (72–99)	91±7 (77–98)	95±3 (88–99)
	Erysipelotrichi*	2±1 (0.1–5)	3±3 (0–10)	3±2 (0–8)
	Bacilli*	1±2 (0–7)	1±1 (0–5)	1±1 (0–4)
Bacteroidetes	% of Total	22±9 (9–37)	21±12 (1–45)	20±11 (3–45)
	Bacteroidia*	52±12 (30–71)	56±18 (28–99)	46±11 (34–71)
	Sphingobacteria*	1±2 (0–7)	1±1 (0–4)	2±2 (0–7)
	Flavobacteria*	2±3 (0.1–12)	4±5 (0–17)	5±4 (0–13)
Proteobacteria	% of Total	3±3 (0.1–15)	2±2 (0.3–6)	2±1 (0–6)
	Alphaproteobacteria*	4±11 (0–50)	8±15^a^ (0–53)	3±3 (0–9)
	Betaproteobacteria*	20±16 (0–67)	19±18 (0–74)	20±26 (0–100)
	Deltaproteobacteria*	6±6 (0–24)	16±19^a^ (0–68)	6±9 (0–32)
	Gammaproteobacteria*	68±22 (21–100)	56±26 (17–88)	63±32 (0–96)
Actinobacteria	Actinobacteria	2±1 (0.1–5)	2±1 (0.4–6)	2±1 (0–5)
Fusobacteria	Fusobacteria	0.1±0.3 (0–1)	0.1±0.1 (0–0.3)	0.3±1 (0–3)
Spirochaetes	Spirochaetes	0.5±0.4 (0.1–1)	0.4±0.4 (0–1)	0.3±0.4 (0–1)
Tenericutes	Mollicutes	0.9±2 (0–7)	1±1 (0–5)	0.8±0.7 (0–2)
Verrucomicrobia	Verrucomicrobiae	0.1±0.2 (0–1)	0.1±0.2 (0–1)	0.1±0.1 (0–1)

* : represents % of phyla.

Values are given as means ± SEM with range.

a <0.05 compared with Control and UC.

**Table 3:** Differently expressed genes (Fold Change >1.5) in biopsies from CD and UC patients as compared with control tissue.

Function	Symbol	Gene	Fold change	
			CD	UC
Cytokine	CSF1	Colony-stimulating factor 1		−1.53
	CSF2	Colony-stimulating factor 2	1.65	1.66
	CSF3	Colony-stimulating factor 3	3.58	3.83
	IFNG	Interferonγ	2.79	
	IL1A	Interleukin 1α	7.50	4.70
	IL1B	Interleukin 1β	5.28	3.51
	IL2	Interleukin 2	1.51	
	IL2RA	interleukin 2 receptor alpha	2.15	
	IL4	Interleukin 4	3.05	6.44
	IL5	Interleukin 5	−1.64	
	IL6	Interleukin 6	2.37	1.59
	IL8	Interleukin 8	3.97	12.60
	IL-13	Interleukin 13	−2.46	−1.56
	IL17	Interleukin 17	2.84	18.61
	LTA	Lymphotoxin alpha	1.65	
	TNF	Tumor necrosis factor	1.61	
Chemokine	CCL2	Chemokine (C-C motif) ligand 2	1.73	
	CCL3	Chemokine (C-C motif) ligand 3	1.62	
	CCL5	Chemokine (C-C motif) ligand 5		−1.98
	CCL19	Chemokine (C-C motif) ligand 19	2.35	1.63
	CCR4	Chemokine (C-C motif) receptor 4	2.57	
	CXCL10	Chemokine (C-X-C motif) ligand 10	5.59	
	CXCL11	Chemokine (C-X-C motif) ligand 11	14.42	2.02
Cellular Marker	CD3E	CD3e molecule, epsilon	−1.58	
	CD8A	CD8a molecule	−1.82	−1.91
	CD40	CD 40 molecule	1.61	
	CD40LG	CD 40 ligand	2.54	
	CD68	CD68 molecule	1.54	
	CTLA4	Cytotoxic T-lymphocyte-associated protein 4	1.91	1.92
	HLA-DRB1	Major histocompatibility complex, class II, DR beta 1	14.42	37.59
	SKI	Sarcoma viral oncogene homolog		−1.59
	TBX21	T-box 21		−1.74
	TNFRSF18	Tumor necrosis factor receptor superfamily, member 18	1.71	
Intracellular Signaling	AGTR1	Angiotensin II receptor, type 1	−2.73	−1.51
	SMAD7	SMAD family member 7		−1.74
Secreted Factors	NOS2A	Nitric oxide synthase 2, inducible	2.05	4.85
Apoptosis	FASLG	Fas ligand	−1.83	−1.95
	BCL2	B-cell CLL/lymphoma 2		−1.57
Enzymes	ACE	Angiotensin I converting enzyme		−1.52
	CYP7A1	Cytochrome P450, family 7, subfamily A	1.57	−2.20
	PTGS2	Prostaglandin-endoperoxide synthase 2	2.29	
Cellular Migration	FN1	Firbronectin 1		−1.51
	ICAM1	Intercellular adhesion molecule 1	1.77	
	SELE	Selectin E	2.66	4.66
	SELP	Selectin P	1.95	1.89
Degranulation, Compliment	C3	Complement component 3	1.82	
	GNLY	Granulysin		−1.51
	PRF1	Perforin 1		−1.72

Other	EDN1	Endothelin 1		−1.66
	TFRC	Transferring receptor	1.53	

#### Ulcerative Colitis

Genes up-regulated under basal conditions included cytokines (IL1A, IL1B, IL4, IL6, IL8, IL17, CSF2, CSF3), chemokines (CXCL11, CCL19), secreted factors (NOS2A), and molecules related to cellular migration (SELE, SELP). Genes down-regulated included cytokines (IL13, CSF1,), chemokines (CCL3, CCL5) and molecules involved in intracellular signalling (SMAD7, BCL2, CYP7AI, AGTR1), and apoptosis (FASLG). These results suggest a heightened inflammatory-type environment in tissue from UC patients, with increased mRNA for pro-inflammatory cytokines, chemotactic factors, cellular markers involved in T cell activation, and adhesion molecules, along with decreased mRNA of markers of apoptosis and cytotoxic T cells. In addition to altered gene expression, UC patients also had changes in mucosal-associated microbiota (Table 2).

#### Crohn's Disease

CD patients exhibited increased expression of several genes related to inflammation, including cytokines (IFNG, IL12RA, IL1A, IL1B, IL4, IL6, IL8, IL17, CSF3, TNF), chemokines (CXCL10, CXCL11, CCR4, CCL19), secreted factors (NOS2A), and molecules related to cellular migration (REN, ICAM1, SELE, SELP). Genes down-regulated included cytokines (IL13), chemokines (CCL5) and molecules involved in intracellular signalling (AGTR1) and apoptosis (FASLG). Microbial analysis revealed that samples from CD patients had altered gut microbiota in comparison with controls and UC patients (Table 2).

### Principal Component Analysis (PCA) and Correlation Matrix

**Figure 1:**
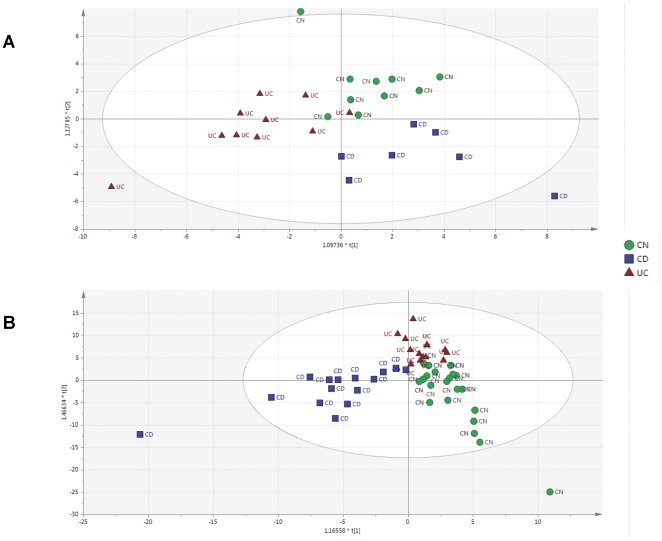
Orthogonal partial least-squares discriminant analysis (OPLS-DA) plot of gene transcripts (A) and microbiota (B) of controls (green circles), UC (red triangles) and CD (blue squares) patients. (A) Network analysis based on 96 differentially expressed genes between groups using an OPLS-DA model showed CD and UC patients to cluster independently from controls. (B) Analysis of mucosal-associated bacteria in snap-frozen biopsies also showed CD and UC patients to cluster independently from controls.

**Figure 2:**
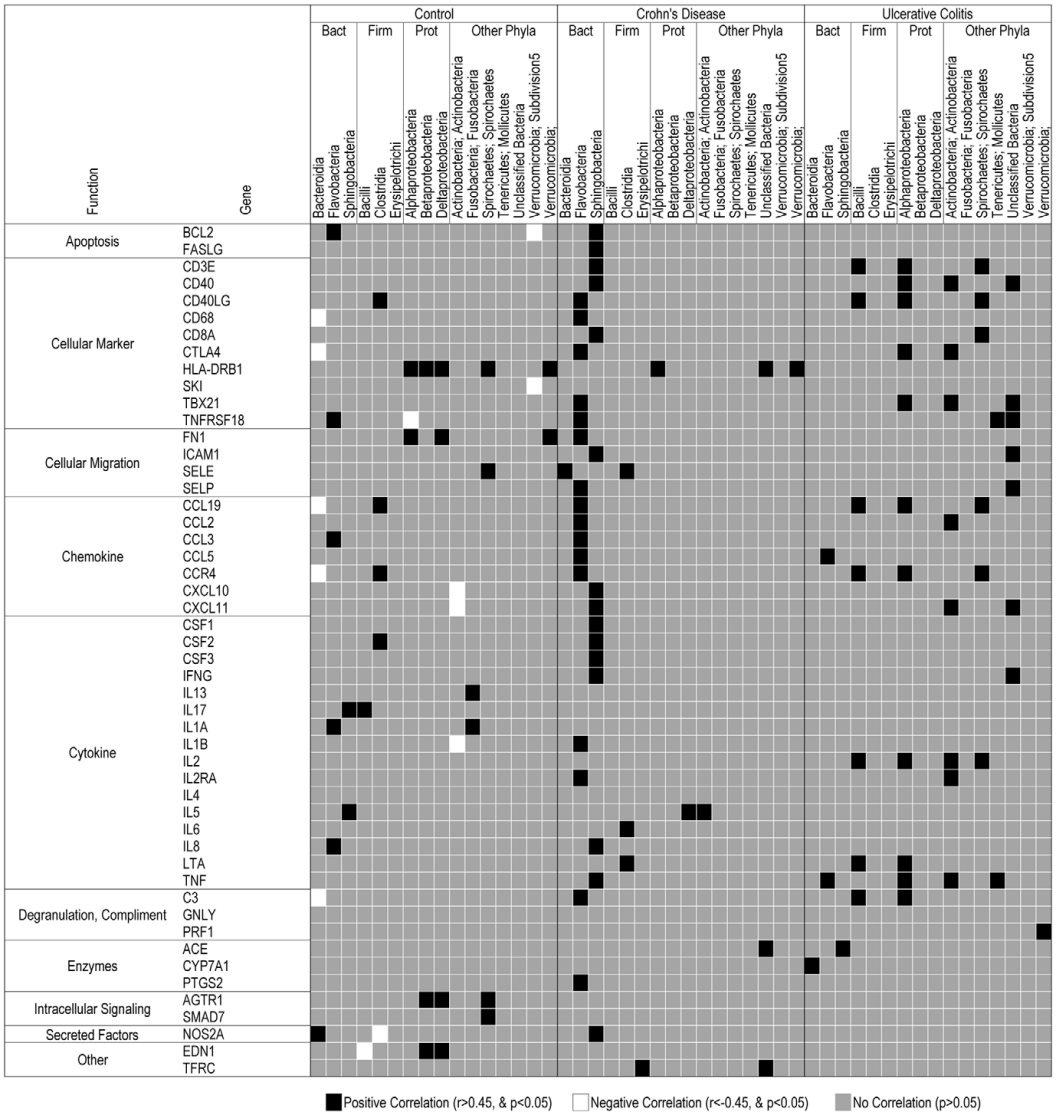
Correlations between microbiota and gene expression showing both positive and negative relationships. Microbes are classified as by phyla and genera. Genes are organized into functional groups. Bact: Bacteroides; Firm: Firmicutes; Prot: Proteobacteria. Black squares represent positive correlations (r>0.45); grey squares show no significant correlation; white squares represent negative correlations (r<0.45).

Orthogonal partial least-squares discriminant analysis (OPLS-DA) of gene expression showed UC patients to cluster independently from CD and controls (Figure 1A) with gene expression of CSF3 (colony stimulating factor 3), IL-17, and HLA-DRB1 primarily driving the separations. OPLS-DA analysis of microbiota also showed CD and UC patients to cluster independently from controls. Both positive and negative correlations between gene expression and specific microbial groups were seen in all groups (Figure 2). However, both CD and UC patients had more positive and less negative correlations as compared with controls. In particular, in CD patients, positive correlations were predominantly found within the Bacteroidetes phyla. These results clearly demonstrate altered microbial-host relationships exist in patients with both CD and UC, and further, these altered relationships exist in the absence of histological disease in patients in clinical remission.

### Gene Expression in Response to Bacterial DNA

Having determined that the gut luminal environment differed between IBD patients and healthy controls, we sought to determine if the patient groups differed in their response to bacterial DNA. Biopsies from an age matched subset of CD (n = 6) and UC (n = 6) patients and controls (n = 6) (Table 4) were cultured for two hours in the presence or absence of bacterial DNA (*S. dublin* or *L. plantarum*) and early changes in gene expression were analyzed (Table 5).

**Table 4:** Clinical parameters for patients in response to bacterial DNA experiments.

Category	Sub-Category	Control (n = 6)	Crohn's Disease (n = 6)	Ulcerative Colitis (n = 6)
Mean Age (yrs) (range)		49.3(18–66)	44.0(23–68)	40.2(24–72)
Gender	Female	3 (50%)	4 (66%)	2 (34%)
	Male	3 (50%)	2 (34%)	4 (66%)
Disease Duration (yrs)		n/a	8 (5–22)	5 (4–8)
Medication	5-ASA	n/a	2 (33%)	5 (83%)
	Steroids		0 (0%)	2 (33%)
	Immunomodulators*		2 (33%)	0 (0%)
	Biologics		1 (16%)	0 (0%)
	Antibiotics		0 (0%)	0 (0%)
	None		2 (33%)	1 (16%)

* Immunomodulators: azathioprine and/or infliximab.

5-ASA- 5-amino salicylic acid.

**Table 5:** Gene expression changes (>1.5 fold) in biopsies from CD and UC patients in comparison with controls in response to bacterial DNA.

Group	Treatment	Increased	Decreased
**Control(n = 6)**	*L. plantarum*	CD19, CYP7AI, LTA, CCR2, CD40LG, IL10	IL4
	*S. dublin*	CYP7A1, CD19, LTA, CCR7, IL5, CCR2, TNFRSF18, CD40LG	IL13
**CD(n = 6)**	*L. plantarum*	REN, IL-17, AGTR1, CYP7A1	IL4, TBX21, IL6, TNFRSF18, IL13, HLA-DRB1, FN1, C3
	*S. dublin*	CCL3, IL-17, IL-1A, CXCL11, CYP7A1, REN, IL1B, IL8	IL4, CCL19, TNFRSF18, CD19, HLA-DRB1, C3, CCR7, IL-13
**UC(n = 6)**	*L. plantarum*	REN, IL-1A, TBX21, AGTR1, IL-4, CXCR3	CCR2, IL5, LTA, IL-12B, FASLG, TNFRSF18, TFRC
	*S. dublin*	CCL19, TNFRSF18, CXCL10, IL1A, AGTR1, CXCL11	CD3E, LTA, IL5

#### Controls

A total of 9 genes showed a ≥1.5 fold change in response to bacterial DNA, with 2 gene responses specific to *L. plantarum* and 4 specific to *S. dublin*. Gene responses similar to both bacterial DNAs included an up-regulation of lymphotoxin α (LTA), CCR2, CD19, and CD40LG. Specific responses to *L. plantarum* included an up-regulation of IL10 and a down-regulation of IL4. Specific responses to *S. dublin* included an up-regulation of IL5, CCR7, and TNFRSF18 and a down-regulation of IL13.

#### Ulcerative Colitis

In UC patients, a total of 20 genes showed a fold change of ≥1.5 to bacterial DNA, with 7 gene responses specific to *L. plantarum* and 4 specific to *S. dublin*. Again, several gene responses were similar to both bacterial DNAs, and included a down-regulation of IL5, LTA, and an up-regulation of AGTR1 and IL1A. Specific responses to *L. plantarum* included a down-regulation of IL12B, CCR2, FASLG, TFRC, and an up-regulation of CXCR3, TBX21, and REN. Specific responses to *S. dublin* included a down-regulation of CD3E and an up-regulation of CCL19, CXCL10, and CXCL11.

#### Crohn's Disease

In CD patients, a total of 18 genes showed a fold change of ≥1.5 to bacterial DNA, with 4 gene responses specific to *L. plantarum* and 8 specific to *S. dublin*. Responses similar to both bacterial DNAs included a down-regulation of IL13, HLADRB1, TNFRSF18, and C3, and an up-regulation of IL17 and REN. Specific responses to *L. plantarum* included a down-regulation of IL6 and IFN1. CD patients were generally more responsive to *S. dublin* compared with either controls or UC patients, with an upregulation of IL1A, IL1B, IL8, CCL3, CXCL11, and a down-regulation of CCL19 and CD19.

### Gene Networks of DNA-treated and Control Biopsies

**Figure 3:**
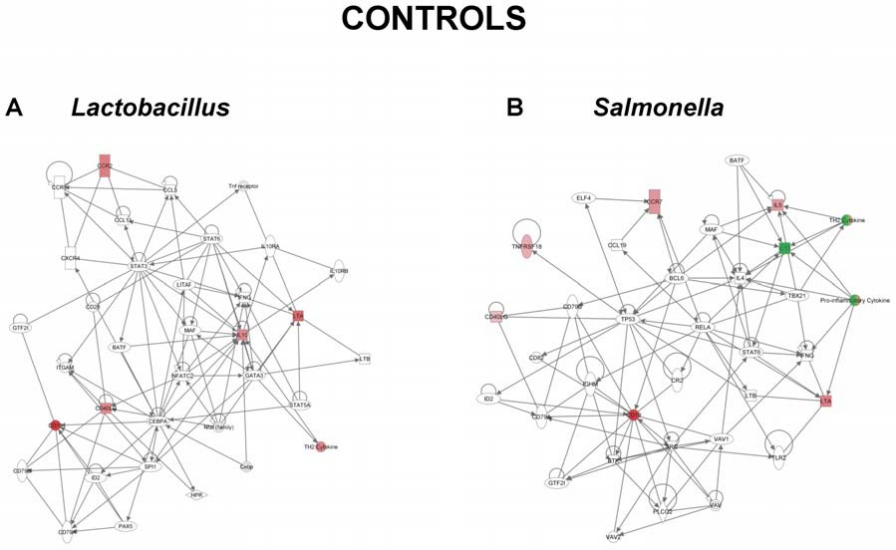
Ingenuity Pathway gene network. The most highly significant gene networks identified in the Ingenuity Pathway analysis of the gene expression data in response to bacterial DNA are shown. Control patient responses to DNA from L. *plantarum* are shown in (A) and to *S. dublin* in (B). Networks are displayed graphically as nodes (genes/gene products) and edges (the biological relationships between the nodes). The intensity of the node color indicates the degree of up (red) or down (green) regulation in gene expression. Nodes are displayed using shapes that represent the functional class of the gene product. Edges are displayed as a direct interaction (solid line).

**Figure 4:**
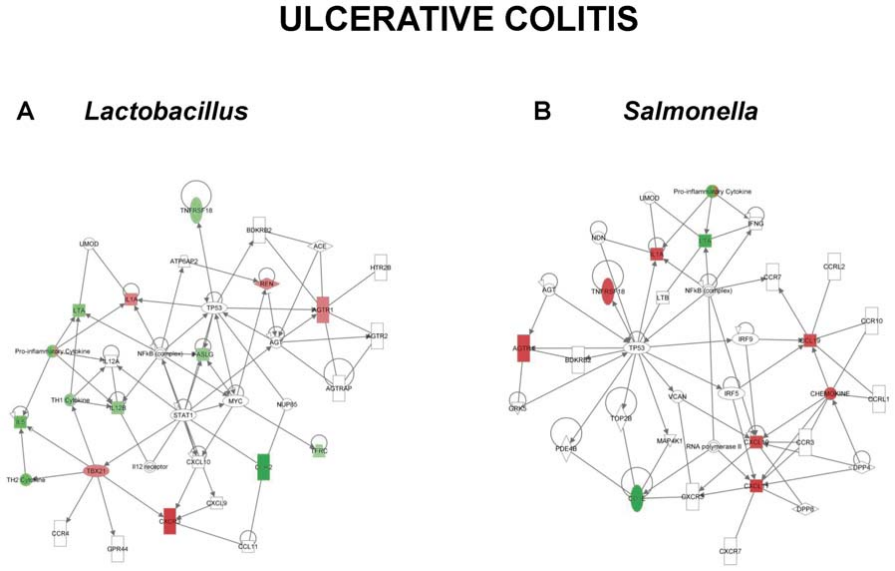
Ingenuity Pathway gene network. The most highly significant gene networks identified in the Ingenuity Pathway analysis of the gene expression data in response to bacterial DNA are shown. UC patient responses to DNA from *L. plantarum* are shown in (A) and to *S. dublin* in (B). Networks are displayed graphically as nodes (genes/gene products) and edges (the biological relationships between the nodes). The intensity of the node color indicates the degree of up (red) or down (green) regulation in gene expression. Nodes are displayed using shapes that represent the functional class of the gene product. Edges are displayed as a direct interaction (solid line).

**Figure 5:**
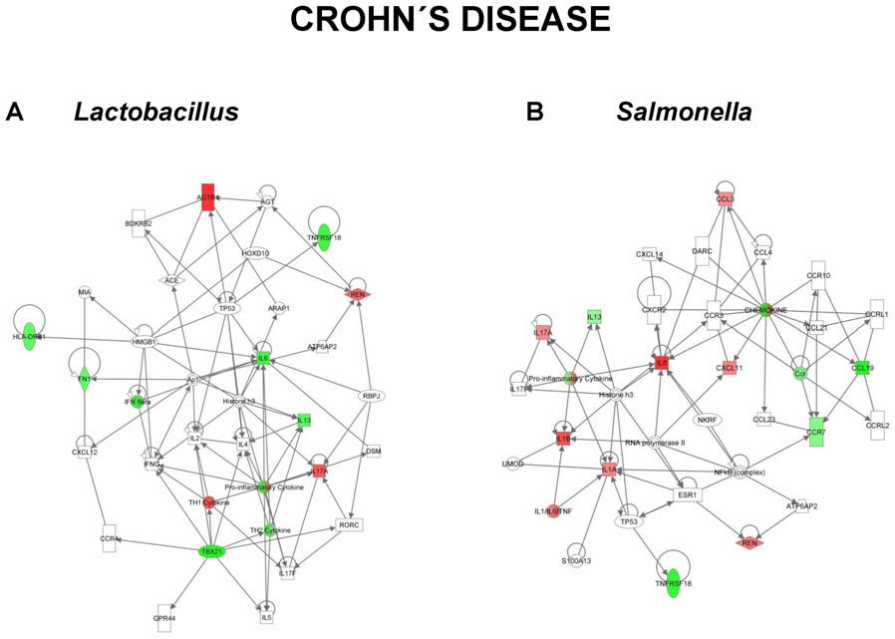
Ingenuity Pathway gene network. The most highly significant gene networks identified in the Ingenuity Pathway analysis of the gene expression data in response to bacterial DNA are shown. CD patient responses to DNA from *L. plantarum* are shown in (A) and to *S. dublin* in (B). Networks are displayed graphically as nodes (genes/gene products) and edges (the biological relationships between the nodes). The intensity of the node color indicates the degree of up (red) or down (green) regulation in gene expression. Nodes are displayed using shapes that represent the functional class of the gene product. Edges are displayed as a direct interaction (solid line).

Alterations in gene expression in the cultured biopsies were entered into the Ingenuity Pathway Analysis (IPA) database and functional networks identified in order to provide biological context to the differentially expressed genes (Figures 3,4,5). This approach allows for changes in gene expression to be related to functional changes within cellular pathways. Early tissue responses to *S. dublin* in control patients (Figure 3) were linked with chemokine and cytokine responses along with NF-κB and STAT6 signaling pathways. In contrast, the response of control patients to *L. plantarum* (Figure 3) involved an up-regulation of IL-10 and involvement of STAT3 and STAT6 pathways, indicative of an anti-inflammatory response. This would be in agreement with our previous studies showing DNA from probiotic strains to have a differential effect on epithelial and immune responses compared with DNA from pathogenic strains [9]. Biopsies from UC patients had a similar response compared with controls to *S. dublin* (Figure 4). Interestingly, in UC patients, *L. plantarum* DNA reduced IL12B and FASLG expression, with an involvement of NF-κB and STAT1 pathways, suggesting that this probiotic strain may have an anti-inflammatory effect in this group of IBD patients (Figure 4). When examining the response of CD patients to *S dublin* DNA, a much different response was seen. As seen in Figure 5, gene networks activated in response to *S. dublin* DNA included the IL17A, IL18, IL6, and IL8 pathways along with NF-κB. Furthermore, the response of CD biopsies to *L. plantarum* DNA did not include any anti-inflammatory pathways, but instead involved an up-regulation of pathways including IL17A and pro-inflammatory cytokines (Figure 5).

### Expression of T and B Cell Markers

As gene expression and cytokine secretion reflect a combined response from both epithelial and immune cells, the changes in expression could be due to altered numbers of immune cells present in the biopsies. However, there were no significant differences between patient groups in expression of the T cell markers, CD4, CD8A and CD3E, the B cell marker, CD19, or the monocyte/macrophage marker CD68 (Table 6). This would suggest that the different responses to bacterial DNA seen in IBD patients likely could not be attributed to population differences in epithelial and immune cells in the biopsies.

**Table 6:** Expression levels of immune cell markers in biopsies from CD and UC patients as compared with controls in response to DNA experiments.

Marker	Target Gene	Group	Fold Change	Δ Expression (Log_10_ RQ)	p-value
T cell	CD3E	CD	−1.58	−0.20	0.39
		UC	−1.25	−0.10	0.69
	CD4	CD	1.42	0.15	0.37
		UC	−1.42	−0.15	0.09
	CD8A	CD	−1.82	−0.26	0.20
		UC	−1.91	−0.28	0.09
B cell	CD19	CD	1.36	0.13	0.59
		UC	−1.23	−0.09	0.76
Monocyte/Macrophage	CD68	CD	1.54	0.19	0.26
		UC	−1.15	−0.06	0.42

### TLR9 and NOD2 Genotyping

Studies have shown TLR9 polymorphisms to be associated with CD and specific TLR9 polymorphisms to be associated with altered TNFα and/or IFNγ levels [14], [15], [16], [17]. In addition, one study has shown interactions between TLR9 polymorphisms and NOD2 variants in patients with CD [18]. In view of these findings, we examined frequencies of these genes to determine if a functional difference in responsiveness to bacterial DNA could be linked with the presence of particular alleles. The genotype frequencies of the TLR9-1237 alleles did not differ between the three groups; however, the rare alleles of both NOD2 SNPs were found more often in the CD patients (Table 7). All frequencies were similar to what has previously been published [18]. No patient had both NOD2 rare alleles or had NOD2 SNP8 and TLR9-1237 SNPs together. Two patients with CD were heterozygotes for the alleles of both NOD2 SNP13 and TLR9-1237 SNPs. There was no apparent relationship between TLR9 polymorphisms and IFNγ gene expression. There was also no difference in the level of TLR9 expression between the groups (data not shown).

**Table 7:** Genotype and allele frequencies of TLR9 and NOD2 polymorphisms.

SNP	Allele	Controls (n = 21)	CD (n = 15)	UC (n = 14)
**TLR9** SNP-1237T/C	TT	15 (71.4%)	11 (73.3%)	10 (71.4%)
	TC	5 (23.8%)	4 (26.7%)	2 (14.3%)
	CC	1 (4.8%)	0	2 (14.3%)
**NOD2** SNP82104C/T	CC	20 (95.2%)	14 (93.3%)	13(92.9%)
	TC	1(4.8%)	1 (6.7%)	1 (7.1%)
	TT	0	0	0
**NOD2** SNP133020insC	C/C	20 (95.2%)	13 (86.7%)	14 (100%)
	C/CC	1 (4.8%)	1 (6.7%)	0
	CC/CC	0	1 (6.7%)	0

## Discussion

In this study, we show that the luminal microenvironment in the transverse colon differs between IBD and control patients in patients in remission and in areas of no histological inflammation. IBD patients had enhanced gene expression of pro-inflammatory cytokines and chemokines and this was associated with a dysbiosis in mucosal-associated microbiota. Correlation analysis showed that IBD patients had increased number of positive correlations between specific gene expression and select microbes compared with controls. Furthermore, this heightened inflammatory environment was associated with altered transcriptional responses to bacterial DNA. In particular, CD patients responded to DNA from both *S. dublin* and *L. plantarum* with enhanced IL17 gene expression. This was in contrast to controls and UC patients, where probiotic DNA stimulated anti-inflammatory pathways and the pathogenic DNA stimulated immune responses. This would suggest that the gut epithelium may have lost the ability to distinguish between bacterial DNA in patients with CD.

In taking of biopsies for study, we were careful to obtain biopsies only in patients who were in remission in order to limit alterations in responses due to active inflammatory processes. However, even in the absence of disease, altered basal gene expression was seen in tissue from both UC and CD patients when compared with controls. In biopsies from CD patients, several Th1-specific IFN-induced chemokines (eg CXCL10, CXCL11) as well as IFNG were up-regulated, indicative of a Th1 type environment. In contrast, IFNG was normal in UC patients, while IL4 was up-regulated, indicative of a more Th2-like cytokine response. Similar to what has previously been shown [19], [20] both CD and UC patients had up-regulated colonic IL17 mRNA expression. Interestingly, IL17 mRNA was actually higher in UC patients compared with CD patients; however, we did not measure IL-17 protein levels to confirm higher IL-17 production in CD patients.

Correlation analysis between gene expression and microbiota demonstrated interesting results with respect to which genes were predominantly correlated with specific microbial taxa. Overall, both IBD groups differed from the control group with increased numbers of positive correlations between particular microbial groups and inflammatory gene expression. The significance of these findings remains to be determined; however, this increased correlation between gene expression and particular microbial communities in IBD patients may reflect an increased exposure of the host immune system to luminal microbes. In addition, the clustering of correlations between numerous genes and Bacteroidetes in the CD patients may represent selective responses in these particular patients. Alternatively, these altered correlations may reflect the presence or absence of particular microbial strains. These results differ from those recently published by Lepage et al [21], who demonstrated a lower number of correlations in patients with UC. However, in their study, they examined a much larger number of genes (21,747) in comparison with our study. In addition, differences in biopsy location (sigmoid vs transverse) may also have contributed.

In epithelial cells, bacterial DNA interacts with TLR9 on the apical or basolateral membrane and both the type of bacterial DNA and the site of interaction can influence cellular responses [8], [9]. Intracellular bacterial DNA can also activate the inflammasome in epithelial and immune cells, resulting in IL-1β and IL-18 secretion [22]. In our experimental system, intact whole biopsies would have included epithelial cells, along with possibly T and B cells, dendritic cells, macrophages, neutrophils, and other innate immune cells. Gene expression measured would therefore be a combination of all cell types and we are not able to differentiate between an epithelial and an immune cell response. However, the advantage to this system is the fact that overall gene expression represents a more physiological response to stimuli than would be seen if only isolated cells were stimulated. Furthermore, by studying whole biopsies, we could somewhat reduce interactions of bacterial DNA with the basolateral surface of epithelial cells, which is known to elicit different responses. In order to determine if the number of immune cells were different between the groups, we measured the expression of specific T and B cell markers, and found no differences. These findings would suggest that the altered gene expression was not likely due to increased numbers of immune cells in the biopsies from IBD patients.

A surprising and interesting finding in these studies was the response of biopsies from patients with CD to DNA from *L. plantarum*. While control responses included the induction of STAT3, which positively regulates IL-10 and maintains epithelial barrier function (15), responses to *L. plantarum* in CD patients included enhanced IL17A expression and gene network analysis suggested an involvement of high-mobility group protein (HMGB). HMGB is released from activated macrophages and monocytes and can drive inflammatory reactions through interactions with TLR4 and the inflammatory receptor RAGE (Receptor for Advanced Glycan Endproducts) [23]. It is interesting that, to date, probiotic therapy has largely failed in CD patients, with one trial using *Lactobacillus rhamnosus* to actually worsen the disease compared with placebo [7]. Findings from this study suggest that immune responses to bacterial DNA appear to be dysfunctional in CD patients, although we cannot differentiate between a failure to properly recognize bacterial DNA by either epithelial or immune cells, or alternatively, a failure to respond appropriately. Overall, regardless of the underlying mechanism, these findings provide further support to the hypothesis that a dysfunctional innate recognition and response to molecules of microbial origin is involved in the pathogenesis of CD in particular.

In these studies we cannot differentiate between TLR9 signaling and inflammasome responses as both may be activated upon exposure to bacterial DNA. The gene encoding for TLR9 is mapped to chromosome 3p21.3 in the vicinity of a shared susceptibility locus for CD and UC. Although one study has shown that the frequency of the 1237 C allele and the C carrier status were significantly increased in CD patients [14], others have shown no difference [24]. In our study, we did not observe a significant increase in this allele in our study population. There was also no association between NOD2 and TLR9 alleles in our population groups as has been shown [18]. Responses to bacterial DNA in our study were not related to the presence of particular TLR9 or NOD2 polymorphisms or to the altered expression of TLR9. Although we did not measure levels of TLR9 protein expression in these studies, it is unlikely that different levels of expression could explain our results, in that CD and UC patients did respond to bacterial DNA, but responded with a different pattern of gene expression. It is also possible that the extensive drug usage that is characteristic of IBD patients could have affected individual host responses; however, in that the IBD patients were all on different types of medication, and the types of medication were similar between the UC and CD patients, it is unlikely that particular drug usage could be the predominant reason for the differential response between UC and CD patients.

In conclusion, patients with IBD have an enhanced level of interaction between gut microbiota and the intestinal epithelium which correlates with dysregulated responses to bacterial DNA, particularly in patients with Crohn's disease. These results suggest that the host response to bacterial DNA may depend not only on the specific type of bacterial DNA encountered, but also on the particular host.

## Supporting Information

Table S1 **Primer Sequences.**
(DOC)
