# Supplementary material for: Patients with Inflammatory Bowel Disease Exhibit Dysregulated Responses to Microbial DNA
Source: PLoS One. 2012 May 23;7(5):e37932. doi: 10.1371/journal.pone.0037932 (PMC3359330; doi:10.1371/journal.pone.0037932)
Supplement: Table S1 — Primer Sequences. (DOC) [file pone.0037932.s001.doc]

**Table S1. Primer sequences**

| SNP | Process Used | Forward Primer 5’-3’ | Reverse Primer 5’-3’ |
| --- | --- | --- | --- |
| NOD2 SNP8 2104C/T (R702W) | PCR | ACC TTC AGA TCA CAG CAG CC | GCT CCC CCA TAC CTG AAC |
|  | SNaPshot | CCC CCC CCC CCC CCC CCT GCC AGA CAT CTG AGA AGG CCC TGC TC | AAA ATC GAT CGA TCC AGC GGG CAC AGG CCT GGC GCC |
| NOD2 SNP13 3020insC (1007fs) | Sequencing | CTC ACC ATT GTA TCT TCT TTT C | GAA TGT CAG AAT CAG AAG GG |
| TLR9 -1237T/C | Sequencing | TTG CCC AGC AAA CAT | GGT CAC ATT TCA GCC CCT AGA |
